# Study on the Adsorption Capacities for Airborne Particulates of Landscape Plants in Different Polluted Regions in Beijing (China)

**DOI:** 10.3390/ijerph120809623

**Published:** 2015-08-14

**Authors:** Wei-Kang Zhang, Bing Wang, Xiang Niu

**Affiliations:** 1 The College of Forestry, Beijing Forestry University, Beijing 100083, China; E-Mails: zhwk123456789@163.com (W.-K.Z.); Wangbing@caf.ac.cn (B.W.); 2 The Research Institute of Forest Ecology, Environment and Protection, Chinese Academy of Forestry, Beijing 100091, China

**Keywords:** aerosol, landscape plants, particulate matter, adsorption capacity, the leaf microstructure morphology

## Abstract

Urban landscape plants are an important component of the urban ecosystem, playing a significant role in the adsorption of airborne particulates and air purification. In this study, six common landscape plants in Beijing were chosen as research subjects, and the adsorption capacities for each different plant leaf and the effects of the leaf structures for the adsorption capacities for particulates were determined. Preliminary results show that needle-leaved tree species adsorbed more airborne particulates than broad-leaved tree species for the same leaf area. *Pinus tabuliformis* exhibits the highest adsorption capacity, at 3.89 ± 0.026 μg·cm^−2^, almost two times as much as that of *Populus tomentosa* (2.00 ± 0.118 μg·cm^−2^). The adsorption capacities for PM_10_ of the same tree species leaves, in different polluted regions had significant differences, and the adsorption capacities for PM_10_ of the tree species leaf beside the Fifth Ring Road were higher than those of the tree species leaves in the Botanical Garden, although the adsorption capacities for PM_2.5_ of the same tree species in different polluted regions had no significant differences. By determining the soluble ion concentrations of the airborne particulates in two regions, it is suggested that the soluble ion concentrations of PM_10_ in the atmosphere in the Botanical Garden and beside the Fifth Ring Road have significant differences, while those of PM_2.5_ in the atmosphere had no significant differences. In different polluted regions there are significant adaptive changes to the leaf structures, and when compared with slightly polluted region, in the seriously polluted region the epidermis cells of the plant leaves shrinked, the surface textures of the leaves became rougher, and the stomas’ frequency and the pubescence length increased. Even though the plant leaves exposed to the seriously polluted region changed significantly, these plants can still grow normally and healthily.

## 1. Introduction

With the rapid development of urbanization and industrialization, urban environmental pollution has become a common concern. This has led to a large amount of research on the urban environmental pollution issue [1,2,3,4,5,6]. Urban air pollution is an ambiguous type of environmental pollution. Among the pollution types, particulate pollution, notably PM_10_ and PM_2.5_ directly or indirectly affects human health. In 1993, Dockery, *et al.* first proposed that particulate matter having an aerodynamic diameter <10 μm (PM_10_) can cause serious harm to human health. With further research, people gradually began to focus on particulate matter having an aerodynamic diameter <2.5 μm (PM_2.5_) and PM_10_) as being harmful to human health. PM_10_ and PM_2.5_, also known as particulate matter, can enter the alveoli through the respiratory system, causing respiratory diseases. They can absorb a large amount of toxic and hazardous substances, and because PM_10_ and PM_2.5_ have large surface area they can cause heart disease and immune system diseases [7,8]. The sources of urban particulate matters are various, and the most significant ones come from the emissions of road traffic, fossil fuel combustion and construction dust [9]. Controlling and reducing urban particulate pollution has become a pressing problem.

Beijing and its energy transport scale have gradually expanded, bringing the average PM_2.5_ concentration in 2013 to 89.8 μg·m^−3^, which is 1.5 times higher than the national standard of 35 μg·m^−3^ [10]. Beijing’s air particulate pollution needs more research, in order to effectively reduce the air particulate pollution problem of the Beijing region. Using plants to absorb atmospheric particle matter has been confirmed as an effective method by domestic and foreign research [11,12,13,14,15], especially coniferous species which can be more effective in removing atmospheric particulates compared to broad-leaved species [16]. A plants’ ability to remove atmospheric particulates mainly relies on its leaf function and leaf structure, such as leaf surface texture, hair, grease and moisture, along with other beneficial features for atmospheric particles absorption, and a huge leaf area supported by a complex stem structure can fix a lot of atmospheric particles. Unfortunately，the atmospheric particles can crimp stomas and reduce the chlorophyll content of leaves, so that gas exchange action is blocked and photosynthesis is decreased [17,18,19,20]. Therefore, analyzing plant stagnation differences with respect to atmospheric particulates in different contaminated areas by the use of plant leaves it is a good method.

The urban forest ecosystem is an important component of the urban ecological system, playing a significant role in the adsorption of pollutants and airborne particulates by increasing the land surface roughness, as well as reducing the wind speed and the adsorptive action of the branches, leaves and stalks [21]. The adsorption capacities for airborne particulates of different tree species display differences, because the different tree species have different crown profiles, branches and leaves ratios and leaf surface characteristics (including waxiness, epidermis, stomates and pubescence length, *etc.*). Both domestic and foreign scholars have embarked on pioneering research on the capacities of urban forestation tree species to improve the environmental quality and adsorb airborne particulates. For example, Smith, *et al.* [22] studied the adsorption capacities for particulates of five coniferous and broadleaved species in England, and the results showed that the adsorption capacity for particulates of *Pinus tabuliformis* is the highest, that of *Sorbus aria* is the second highest and that of *Populus deltoides* is the lowest. Meanwhile, some scholars have elucidated the mechanisms of particulate adsorption of different tree species from the angle of the leaf structures. For example, Pal, *et al.* [23] studied the microscopic morphologic structure changes of the leaves of eight plants in India at different pollution levels, and the results showed that, under serious pollution conditions, the waxiness thickness, pubescence length and stomates density change significantly, which makes them more adaptive in a seriously polluted environment. However, there is little research comparing the adsorption capacities for different particulate size between different tree species and the effect on the leaf morphological structures in different polluted regions.

In this paper, we studied the adsorptive capacities for airborne particulates of the leaves and the mechanisms of six main landscape tree species in different polluted regions in Beijing. The main purpose was to (1) provide a comprehensive study of the most effective use that can be made of trees for improving the air quality in different polluted regions; (2) to analyze the causes of the adsorption capacities for particulates between different tree species from the angle of the leaf structures; and lastly (3) to understand the effect of solution ions on the tree species leaf adsorbing particulars.

## 2. Methods

### 2.1. Study Sites

**Figure 1:**
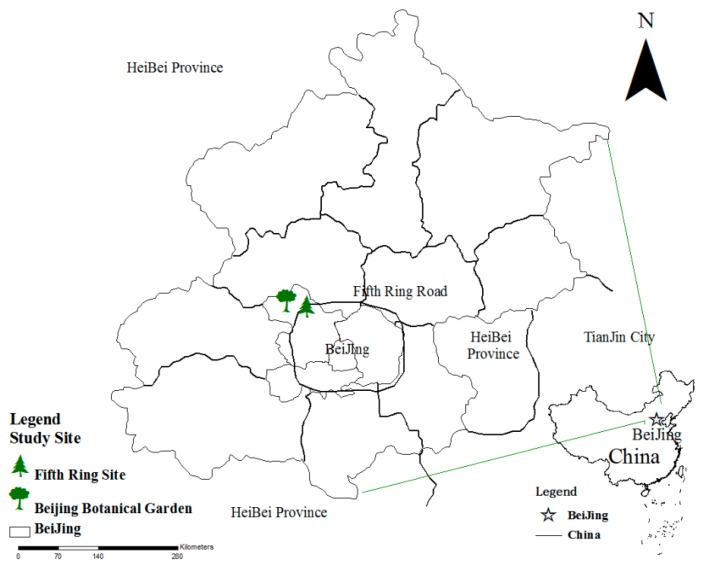
Location of the study sites.

The tree species in this study are from Beijing Botanical Garden and the Fifth Ring Road of Beijing (Figure 1). The Beijing Botanical Garden, located at the foot of Western Hills of Beijing outside the Fifth Ring Road, has a total planning area of 400 hm^2^ and over 1.5 million plants in the garden. It is a comprehensive botanical garden with an integration of exhibitions and protection of plant resources, scientific research, popularization of science, recreation and development. The main tree species include *Pinus tabuliformis, Pinus bungeana, Platycladus orientalis, Pinus koraiensis, Ginkgo biloba*, *Robinia pseudoacacia*, *Buxus megistophylla*, *Populus tomentosa*, *Salix matsudana*, *etc.*, with a vegetation coverage of over 80%. The growth process of the plants in the garden is more greatly affected by human walking and leisure activities than by the consequences of human productive activities such as automobile exhaust and factories. Another selection of tree species in the study is from the greening tree species beside the North Fifth Ring Highway of Beijing, where the growth of the tree species is greatly affected by human productive activities such as automobile exhaust and factories.

The pollution situation of different sites from June to August is shown in Table 1. This shows that the pollution level of the Botanical Garden is lower than that of the Fifth Ring Road. Thus, the Botanical Garden was chosen as the slightly polluted region and the Fifth Ring Road was chosen as the seriously polluted region in this paper.

**Table 1:** PM_2.5_ concentrations of the Botanical Garden and the 5^th^ Ring Road from 6 to 8 months.

PM_2.5_ Concentration/μg·m^−3^	Botanical Garden				Fifth Ring Road			
	June	July	August	Total	June	July	August	Total
<50	18d	12d	19d	49d	15d	10d	17d	42d
50–100	8d	10d	8d	26d	10d	12d	8d	30d
100–150	4d	3d	3d	10d	5d	3d	4d	12d
>150	-	6d	1d	7d	-	6d	2d	8d

Notes: The data are obtained from the air quality monitoring stations of the Beijing Botanical Garden and the Ministry of Environmental Protection of Beijing.

### 2.2. Materials

From June 2014 to August 2014, six species of leaves were collected both in the Beijing Botanical Garden and beside the Fifth Ring Road on a breezeless and sunny day. The six species are as follows.

*Pinus tabuliformis*: this is an effective tree species for the control of environmental pollution due to its evergreen nature, high leaf surface area and the order of the leaves. For this study, two needle-leaved trees were chosen, of which *Pinus tabuliformi* has been proven to have strong adsorptive capacities for airborne particulates, and meanwhile it is also a common landscape plant in northern cities.

*Pinus bungeana*, is another needle-leaved tree species, and one of the endemic tree species of China, has already become a fine tree species in North China, used for garden forestation due to its barren land and drought resistance, wide distribution, good adaptability, various and distinct shapes.

*Populus tomentosa*, is a tall arbor, and a common tree species in the north. *Populus tomentosa* is able to improve the urban ecological system and greatly improve the urban local air quality due to its fast growth rate, good adaptability and wide distribution.

*Ginkgo biloba*, is a gymnosperm medium-sized broad-leaved deciduous tree, and an endemic tree species of China, and also the main tree species used for street greening in Beijing. The leaves of *Ginkgo biloba*, like fans, are smooth with no pubescence, and with poor adsorptive capacities for particulate material [24].

*Acer truncatum*, is a medium-sized tree species, with a dense crown and complex structure, and has strong adsorptive capacities for airborne particulates [25].

*Salix matsudana*, is a medium-sized linear lance late leaf deciduous tree species, and is often used for ornamental plantations.

This project was conducted based on Forestry Standards “Observation Methodology for Long-term Forest Ecosystem Research” of People’s Republic of China. Leaf samples were collected from each tree on the same day in June 1 to August 31 2014 after a period of 10 days without rain. The number of leaves collected for each tree was varied according to the size of each species, to ensure that the total area of each sample was similar. Details are given in Table 2. The leaves from the six tree species were selected at 2–3 m above the ground from individual trees of each species at each site. The leaves in the four dimensions (north, south, east and west) from each tree species were selected. There were three replicates. The selected leaves were mature, complete and healthy. After cutting, leaf samples were immediately transported to the laboratory and analyzed.

**Table 2:** Growth parameters of the six plant species and number of the collected leaves.

Tree Species	Botanical Garden			Fifth Ring Road		
	Height/m	Diameter/cm	Leaf Number/g	Height/m	Diameter/cm	Leaf Number/g
*Pinus tabuliformis*	5.50 ± 1.00	10.15 ± 0.23	100	4.00 ± 0.50	9.52 ± 0.34	100
*Pinus bungeana*	5.00 ± 1.50	10.22 ± 0.42	100	4.50 ± 1.50	8.23 ± 0.42	100
*Populus tomentosa*	15.00 ± 2.00	15.5 ± 0.15	200	13.00 ± 1.50	14.35 ± 0.25	200
*Ginkgo biloba*	12.50 ± 1.50	12.34 ± 0.22	150	10.50 ± 0.50	10.50 ± 0.22	150
*Acer truncatum*	6.50 ± 0.50	10.67 ± 0.13	150	5.50 ± 0.50	9.35 ± 0.13	150
*Salix matsudana*	12.50 ± 1.50	13.54 ± 0.08	150	11.00 ± 0.50	12.79 ± 0.08	150

### 2.3. Adsorptive Amount for Dust per Unit of Leaf Area

The leaves of the different test tree species were put into the feeding box of the aerosol regenerator (QRJZFSQ-I), and by the wind erosion principle, the adsorbed particulates on the leaves were blown off and mixed up to regenerate aerosols. This was repeated for three different replicates for each tree species. Then the mass of TSP, PM_10_ and PM_2.5_ was determined by a DUSTMATE handheld environmental dust detector, which was connected to the aerosol regenerator.

The leaves of the broad-leaved tree species which had been tested were scanned by a scanner (Canon LIDE 110), and then the images were processed by the Adobe Photoshop software, and the leaf area S (unit: cm^2^) was calculated using the leaf area analysis software for. Meanwhile, the leaf areas of the needle-leaved tree species were calculated from the length and diameter measured by a Vernier caliper. The calculation formula for the amount for particulates adsorption per unit of leaf area of different tree species is as follows:

(1) $$Mi=\overset{n}{\underset{1}{\sum }}\frac{m_{ij}}{S_{i}}\;$$

where *M* represents the mass of the adsorbed particulates by leaf area of different tree species (unit: μg·cm^−2^), *i* represents different tree species, and *j* represents the number of particulate species, n = 3 represents different tree replicates. S represents the leaf areas (unit: cm^2^). *mij* represents the mass of TSP, PM_10_ and PM_2.5_ (unit: μg )in the aerosol regenerator (QRJZFSQ-I)

### 2.4. Microstructure of Leaves

(1) The leaves of each tree species in good growth conditions were collected and sealed in a plastic bag immediately, and then taken back to the laboratory; (2) the fresh leaves were cut into small 4 × 4 mm cubes from both sides in the middle of the leaves, which were fixed in 2.5% (volume fraction) glutaraldehyde solution; in the laboratory; (3) after the samples were sprayed with Conductive Coating, the surface structure of the leaves were observed with a S-3400 scanning electron microscope (Hitachi, Tokyo, Japan), and the images were taken with an appropriate scaling [26].

### 2.5. Chemical Analysis of Particulates

The wash solutions were subjected to ion analysis to account for water-soluble particles. The ions measured were those that are listed by the Department of the Environment as the most significant contributors to pollution in Beijing. From June to August in 2014, samples for PM_10_ and PM_2.5_ mass concentrations in the atmosphere were collected with an XHPM2000E Environmental Airborne Particulates Automatic Sampling Instrument in the two experimental regions, with a air flow of 16.7 L·min^−1^ and an interval of 1 hour. The collected sampling filter membranes were taken back to the laboratory, and were extracted with deionized water using ultrasonic agitation. After dilution to a constant volume, 10 kinds of ions (F^−^, HCOO^−^, Cl^−^, NO_3_^−^, SO_4_^2−^, Na^+^, NH_4_^+^, Mg_2_^+^, Ca_2_^+^, K^+^) in the samples were analyzed for concentration analysis with a DX100 Type High Precision Ion Chromatograph (Dionex, Sunnyvale, CA, USA).

Statistical testing was done with the IBM SPSS 19.0 program (SPS Inc., Chicago, IL, USA). Homogeneity of the variances were tested with Levine’s test. The main effects of different species, different pollution area, ions and their interaction were tested with GLM repeated-measures ANOVA. Main effects and contrasts were considered statistically significant when *p* < 0.05.

## 3. Results and Discussion

### 3.1. Results

#### 3.1.1. Differential Analysis of the Adsorptive Capacities of Different Tree Species

**Figure 2:**
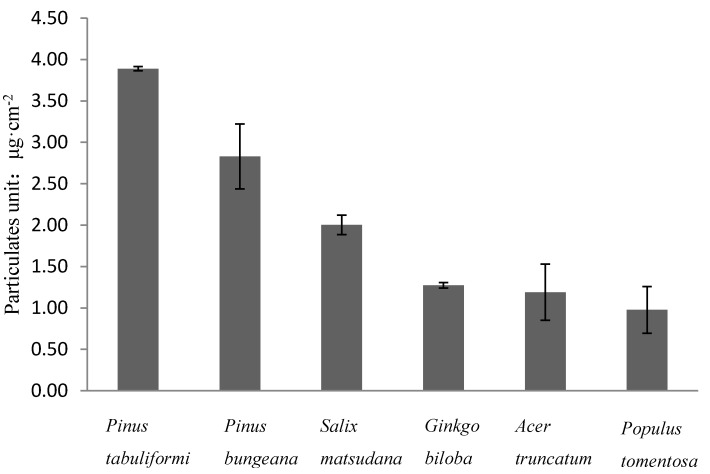
Adsorption the amount of particulate matter in different tree species. Vertical bars represent ± standard error; n = 3. Statistical analysis by 2-way ANOVA between different trees revealed that the differences were significant (*p* < 0.05).

Figure 2 depicts the difference between the adsorptive capacities for particulates per unit of leaf area of different tree species. As shown in the ANOVA results in Figure 2, there is significant difference of the adsorptive capacities for particulates per unit of leaf area of different tree species, and the adsorptive capacities for airborne particulates of different leaves follow the order *Pinus tabuliformis* > *Pinus bungeana* > *Salix matsudana* > *Acer truncatum* > *Ginkgo biloba* > *Populus tomentosa*, among which the adsorptive capacity for particulates per unit of leaf area of the *Pinus tabuliformis* (3.89 ± 0.026 μg·cm^−2^) is the highest, that of *Pinus bungeana* (2.82 ± 0.392 μg·cm^−2^) is the second highest, and that of *Populus tomentosa* is the lowest, at 2.00 ± 0.118 μg·cm^−2^. The adsorptive capacity for particulates per unit of leaf area of *Pinus tabuliformis* is 1.94 times of that of *Populus tomentosa*. As can be seen from the comparison of the adsorptive capacities for airborne particulates of needle-leaved and broad-leaved tree species, the adsorptive capacity for particulates per unit of leaf area of needle-leaved tree species is higher than that of broad-leaved tree species, which is similar to the research results of the adsorptive capacities for airborne particulates on the leaves of different tree species from Norway and Finland reported by Sæbø, *et al.* [27], whose main result was the conifers may be the best choice for pollution-control planting.

**Figure 3:**
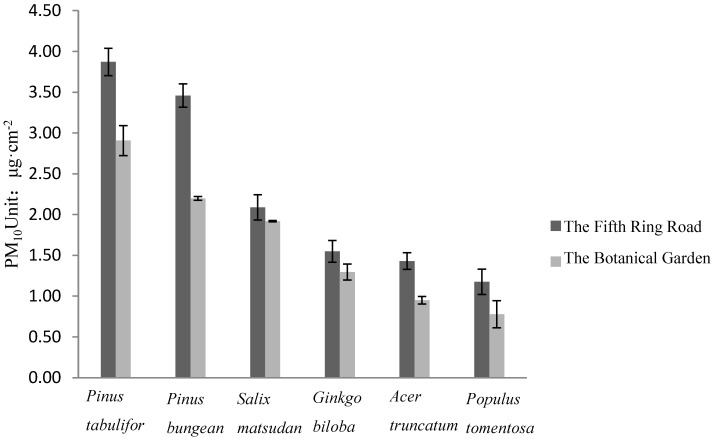
Adsorption PM_10_ diversity of species per unit leaf area in different contaminated areas. Adsorption Vertical bars represent ± standard error; n = 3. Statistical analysis by 2-way ANOVA between interaction different trees and two sites revealed that differences between the two sites were significant (*p* < 0.05), as were differences between species (*p* < 0.05). Interaction between site and species wan found to be non-significant.

Figure 3 depicts the difference in the adsorptive capacities for PM_10_ per unit of leaf area of the six species from the different study sites. As can be seen from the ANOVA, the adsorption of PM_10_ per unit of leaf area of the trees with the Fifth Ring Road is more than that of the same tree leaves in the Botanical Garden.

**Figure 4:**
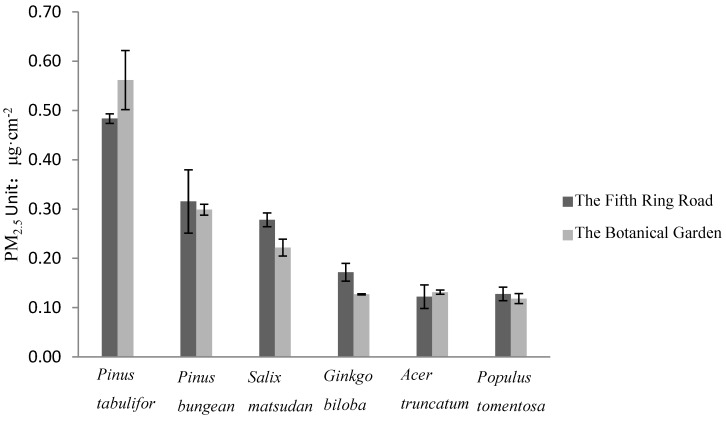
Adsorption PM_2.5_ diversity of species per unit leaf area in different contaminated areas. Vertical bars represent + standard error; n = 3. Statistical analysis by 2-way ANOVA between interaction different trees and 2 sites revealed that differences between the 2 sites were not significant. However, differences between species were found to be highly significant (*p* < 0.05) Interaction between site and species wan found to be not significant.

It is also shows that the ranking of the adsorptive capacities for PM_10_ of different tree species leaves is the same as the rank result in Figure 2. The ANOVA results from Figure 4 show the differences in the adsorptive capacities for PM_2.5_ per unit of leaf area of the trees beside the Fifth Ring Road and the Botanical Garden, and it was found that there was no significant difference between the adsorptive capacities for PM_2.5_ and the rank of the adsorptive capacities for PM_2.5_ of the trees is not consistent with the rank in Figure 1.The rank of the adsorptive capacities for PM_2.5_ of the trees in study is *Pinus tabuliformis* > *Salix matsudana* > *Pinus bungeana* > *Ginkgo biloba* > *Acer truncatum* > *Populus tomentosa*.

#### 3.1.2. The Effect on the Adsorptive Capacities for Particulates of the Leaf Surface Morphological Structures

**Figure 5:**
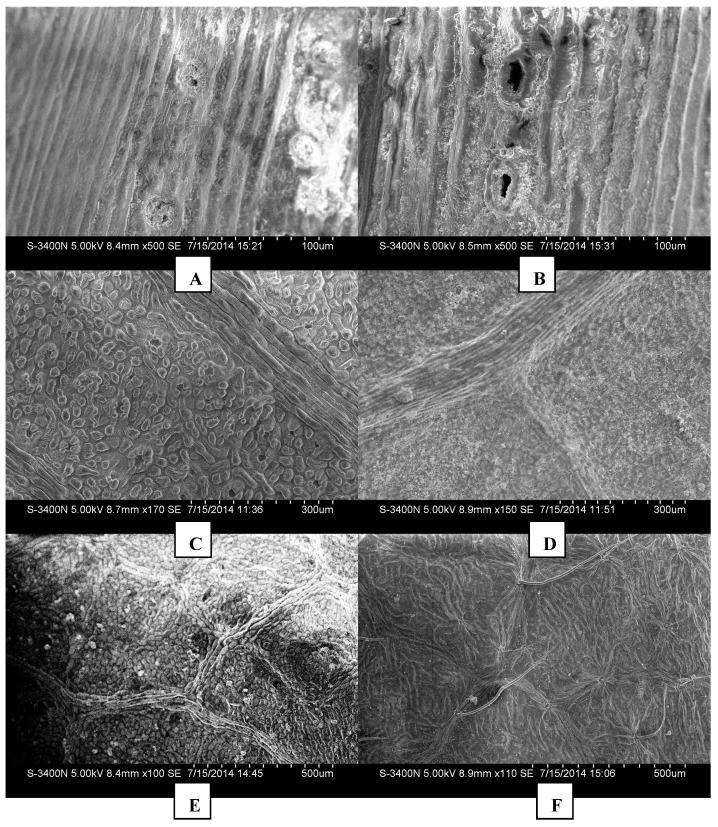
Leaves microcosmic structure of different tree species, (**A**): *Pinus tabuliformis*,(**B**): *Pinus bungeana*, (**C**): *Ginkgo biloba*, (**D**): *Populus tomentosa*, (**E**): *Acer truncatum*, (**F**): *Salix matsudana.*

Figure 5 shows the leaf surface microstructures for the six species under study. The stomatic arrangement density and surface roughness of *Pinus tabuliformis* and *Pinus bungeana* are higher than that of *Populus tomentosa*, *Ginkgo biloba*, *Acer truncatum* and *Populus tomentosa*.

The stomas of *Pinus tabuliformis* (A) are circular and arranged vertically, have higher density and secrete around them, adsorption of irregular particulates on them. This helps dust particulates to stay close to the stomas on the leaf surfaces due to the irregular cell arrangement, and the stomas become the most concentrated location for dust. The leaf texture is arranged closely, with raised bars, rough and irregular surface. *Pinus bungeana* (B), has a rough surface and oval stomas, which are larger than those of *Pinus tabuliformis* under the same microscopic magnification. However, *Pinus bungeana* is smoother around the stomas than *Pinus tabuliformis* and has less adsorbed particulates. *Pinus bungeana* has an irregular texture with sheet distribution and no observable pubescence, and has waxes on the leaf surface. *Ginkgo biloba* (C) has a smooth surface and low stoma density, with a clear and distinct stoma, with no waxes and no epidermal cilium. The epidermis of *Populus tomentosa* (D) tends to be smooth, the stomas are very small, sunken under the corneum, and are completely surrounded by the corneum arch covering protuberances, with no secretion. The texture is clear and distinct with shallow mesh ornamentation, with no epidermal pubescence and glands and only a few adsorbed particulates on the surface. *Acer truncatum* (E) has a radial and parallel distribution of its stomas, with some shallow ridges, and has similar mesh or honeycomb trench organizations (E2), with clear, distinct but irregular texture. *Salix matsudana* (F) has larger and smoother stomas, with lower density and smaller opening of the stomas, and no obvious fluctuation. Around the stomas, *Salix matsudana* has intensive and shallow linear decoration and more surface cilia, which are soft and long and short cylindrical, arranged sparsely.

#### 3.1.3. Effect on the Leaf Structural Morphology in Different Polluted Regions

**Figure 6:**
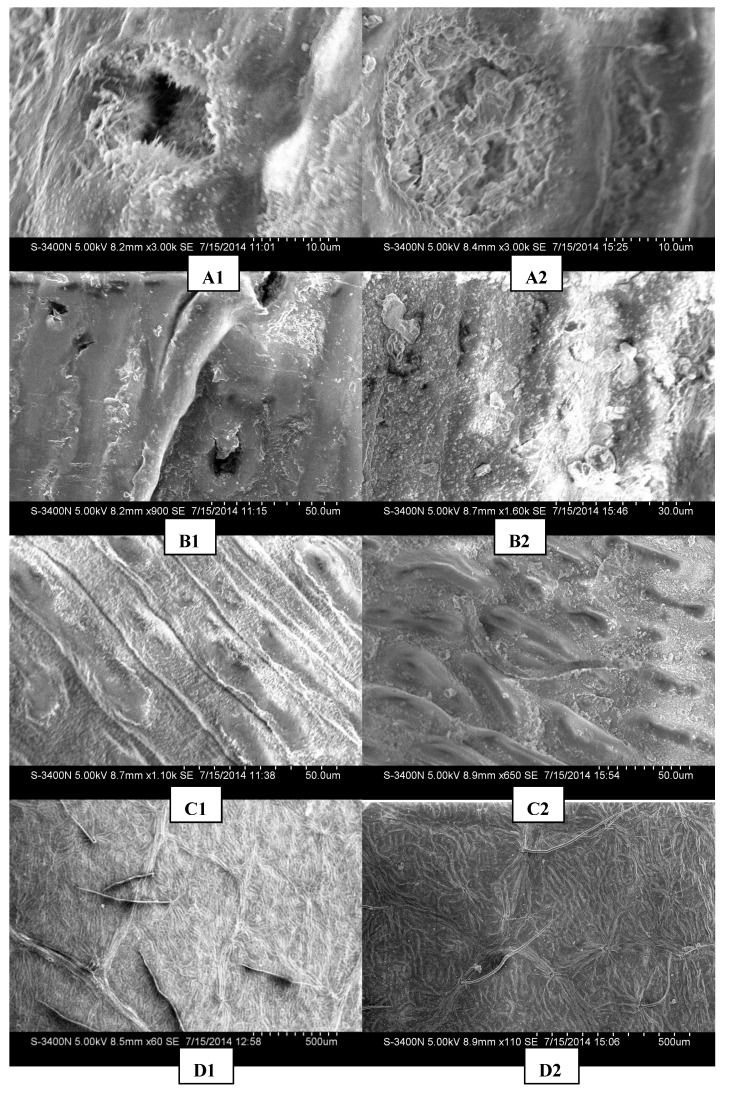
Different impacts on leaf morphology of the contaminated area.

Figure 6 shows the morphological characteristics of the plant leaf structures in different polluted regions. As shown in Figure 6, significant changes of plant leaf microstructures occur in seriously polluted regions:

*Changes of stomas* (A1, A2): compared to the slightly polluted region (the Botanical Garden, A1), the polluted region (the Fifth Ring Road, A2) shows the cells around the plant leaf stomas to collapse and melt, with particulates all around the stomas, closed or half closed.

*Changes of wax coat* (B1, B2): in the seriously polluted region, the wax coat on the plant leaves disappears gradually (B2), for the adsorption for more airborne particulates. Compared to B1, B2 shows more adsorbed particulates.

*Changes of surface texture* (C1, C2): in seriously polluted region, the plant leaf texture becomes more irregular and rougher, so that the contact angle of the leaf surface becomes larger, which makes the adsorption for particulates more effective [28].

*Changes of leaf pubescence* (D1, D2): the plant leaf surface pubescence becomes longer and softer in seriously polluted region, but the density of the pubescence decreases accordingly.

#### 3.1.4. Elemental Composition Analysis of Particulates

Table 3 shows the determination results of the soluble ion concentrations of airborne particulates in the two observation points of the Botanical Garden and the Fifth Ring Road from June to August.

**Table 3:** Determination Results of the Soluble Ion Concentrations of Airborne Particulates in Different Polluted Regions from June to August in 2014 (mean + SE in μg·cm^−3^).

Particulates	Regions	F^−^	HCOO^−^	Cl^−^	NO_3_^−^	SO_4_^2−^	Na^+^	NH_4_^+^	Mg^2+^	Ca^2+^	K^+^	Total	*P* ^*^
**PM_10_**	the Botanical Garden	0.04 ± 0.02	0.12 ± 0.02	1.19 ± 0.05	2.19 ± 0.05	9.37 ± 0.03	4.51 ± 0.02	0.28 ± 0.01	0.24 ± 0.06	1.45 ± 0.02	0.31 ± 0.01	19.70 ± 1.20	>0.05
	the Fifth Ring Road	0.08 ± 0.02	0.77 ± 0.03	1.82 ± 0.02	9.17 ± 0.03	13.70 ± 0.10	14.14 ± 0.05	0.47 ± 0.01	0.09 ± 0.01	0.26 ± 0.01	0.38 ± 0.01	40.87 ± 2.30	
**PM_2.5_**	the Botanical Garden	0.04 ± 0.01	0.18 ± 0.01	1.38 ± 0.05	9.60 ± 0.06	21.65 ± 0.50	8.42 ± 0.08	2.41 ± 0.02	0.32 ± 0.02	1.62 ± 0.03	0.92 ± 0.02	46.54 ± 2.10	<0.05
	the Fifth Ring Road	0.04 ± 0.01	0.36 ± 0.02	1.16 ± 0.04	7.28 ± 0.11	16.10 ± 0.70	12.14 ± 0.07	0.28 ± 0.04	0.05 ± 0.01	0.07 ± 0.06	0.23 ± 0.03	37.70 ± 1.30	

^*^ represent mean+ standard error; n = 3; Statistical analysis by 2-way ANOVA between interaction ions and 2 sites revealed that Soluble Ion Concentrations of PM_10_ between the 2 sites were not significant (*p* > 0.05). However, PM_2.5_ between 2 regions were found to be highly significant (*p* < 0.05).

Table 3 shows that the ion concentrations of airborne particulates beside the Fifth Ring Road are higher than the ion concentrations of airborne particulates in the Botanical Garden, although the main ion compositions of airborne particulates in the two regions are almost similar. The main ions of the airborne particulates at the Botanical Garden and the Fifth Ring Road sites are SO_4_^2−^, NO_3_^−^, Na^+^, respectively. This shows that the main sources of airborne particulates in the experimental regions from June to August were automobile exhaust, the Earth’s crust and secondary sources. However, the total ion contents of particulates in different polluted regions have differences, including the ion concentrations of PM_10_ in the atmosphere in the Botanical Garden and within the Fifth Ring Road have great differences, 19.70 μg·cm^−3^ in the Botanical Garden and 40.87 μg·cm^−3^ within the Fifth Ring Road, a difference of 21.18 μg·cm^−3^, which indicates that the ion concentrations of PM_10_ in the atmosphere within the Fifth Ring Road are much higher than those in the Botanical Garden, while the ion contents of PM_2.5_ in the two regions have little differences, 46.54 μg·cm^−3^ in the Botanical Garden and 37.70 μg·cm^−3^ within the Fifth Ring Road. The difference is only 8.84 μg·cm^−3^, which indicates that the ion concentrations of PM_2.5_ in the two regions of the Botanical Garden and the Fifth Ring Road are basically the same, which indicates from another aspect—that the adsorption capacities for PM_10_ of trees species in the Botanical Garden and within the Fifth Ring Road have significant differences, while the adsorption capacities for PM_2.5_ of trees species in the two regions have no differences.

### 3.2. Discussion

Although the trees were located in different pollution areas, the same species showed no different adsorptive capacities for PM_2.5_ particulates. As would be expected because of their proximity to a rich particle source, more PM_10_ particulate material was captured by trees at the polluted, rather than at the background site. Due to their size and weight, the PM_2.5_ particulate material is smaller in size and lighter than PM_10_, the PM_10_ particles settle out or are impacted onto leaf surfaces much closer than do PM_2.5_ particles [29]. As a result, the PM_2.5_ material has a much greater residence time in the atmosphere. This means that it can travel greater distances, leading to more even spread of PM_2.5_ particle concentrations far from their source [30]. Because the ambient concentrations of PM_2.5_ particles should be similar at both sites (Table 1), the Botanical Garden site is located on the exposed and windy south Downs. It is precisely seen that PM_2.5_ particles are captured with similar efficiency at both study sites.

All trees captured the two size ranges of particulate matter (PM_10_ and PM_2.5_) with similar efficiency at both study sites, it is that the adsorptive capacities of the needle-leaved tree species as a whole are higher that of the broad-leaved tree species, which is consistent with the research results of Qi, *et al.* [31] for which the main reasons are that the leaves of different tree species have different adsorptive capacities for dust due to their different morphological structure, and needle-leaved tree species have rougher surface compared to broad-leaved tree species, can secrete grease, and have higher stomate number arrangement density. These aspects make more particulates adsorb on the surface of leaves, and thus increase the adsorptive capacities of needle-leaved tree species. In this study, *Pinus tabuliformis* ranked first, but all these species have the potential ability to capture particulate matter as can be inferred by seeing the structural morphology of the leaves. Among the broad-leaf trees, *Salix matsudana* has high value for particulate capture, which can well be explained by the species’ rough and hairy abaxial leaf surfaces. In the seriously polluted region, the effects on the leaf microstructure are that the stomas are blocked or half blocked by particulates, the cells around the stomas collapse, and the pubescences become longer and softer. Chai, *et al.* [20] found, as the plant leaf pubescence becomes longer and thinner, so it’s easier to adsorb particulates and more difficult for them to escape, consequently, the adsorptive effect for dust increases. In order to adapt to the environment for the growth, these changes speed up the metabolism [21]. Meanwhile, Pal, *et al.* [23] found that plant leaf pubescence becomes longer and leaf texture becomes rougher in seriously polluted scenarios, which is the strategy of the plant to the seriously polluted environment and makes the plant adapt to the changes of the environment better [32,33,34].

The relationship between concentration of sodium and sulphate ions in the particles show that in this study the major component of the PM_10_ and PM_2.5_ particles was Na_2_SO_4_.Because Beijing is a developing international metropolis, which had almost 23 million and one in four people have a car. It is suspected that automobile exhaust and the Earth crust would make significant contributions to the concentration of these ions, particularly for the PM_2.5_ particulates.

## 4. Conclusions

The main conclusions that can be drawn from the presented study are as follows:

Trees can capture significant quantities of particles from the atmosphere, especially PM_2.5_ particles, with the potential to improve local air quality. The ability to capture PM_2.5_ particles with the same tree species is similar .even in different pollution areas. The leaf structural morphology is thought to have animportant impact on adsorb of the particles from atmosphere. Future research will attempt to remove the urban airborne particulates, to manage the urban environment and to improve the environmental quality by increasing the urban vegetation coverage [35,36].
